# Implicit Processing of Pitch in Postlingually Deafened Cochlear Implant Users

**DOI:** 10.3389/fpsyg.2019.01990

**Published:** 2019-09-11

**Authors:** Barbara Tillmann, Bénédicte Poulin-Charronnat, Etienne Gaudrain, Idrick Akhoun, Charles Delbé, Eric Truy, Lionel Collet

**Affiliations:** ^1^CNRS UMR5292, INSERM U1028, Auditory Cognition and Psychoacoustics Team, Lyon Neuroscience Research Center, Lyon, France; ^2^University of Lyon, Lyon, France; ^3^Université Claude Bernard Lyon 1, Villeurbanne, France; ^4^LEAD-CNRS, UMR5022, Université Bourgogne Franche-Comté, Dijon, France; ^5^University Medical Center Groningen, University of Groningen, Groningen, Netherlands; ^6^School of Psychological Sciences, The University of Manchester, Manchester, United Kingdom; ^7^CNRS UMR5292, INSERM U1028, Brain Dynamics and Cognition Team, Lyon Neuroscience Research Center, Lyon, France

**Keywords:** music perception, cochlear implants, implicit investigation method, auditory sensory memory, priming

## Abstract

Cochlear implant (CI) users can only access limited pitch information through their device, which hinders music appreciation. Poor music perception may not only be due to CI technical limitations; lack of training or negative attitudes toward the electric sound might also contribute to it. Our study investigated with an implicit (indirect) investigation method whether poorly transmitted pitch information, presented as musical chords, can activate listeners’ knowledge about musical structures acquired prior to deafness. Seven postlingually deafened adult CI users participated in a musical priming paradigm investigating pitch processing without explicit judgments. Sequences made of eight sung-chords that ended on either a musically related (expected) target chord or a less-related (less-expected) target chord were presented. The use of a priming task based on linguistic features allowed CI patients to perform fast judgments on target chords in the sung music. If listeners’ musical knowledge is activated and allows for tonal expectations (as in normal-hearing listeners), faster response times were expected for related targets than less-related targets. However, if the pitch percept is too different and does not activate musical knowledge acquired prior to deafness, storing pitch information in a short-term memory buffer predicts the opposite pattern. If transmitted pitch information is too poor, no difference in response times should be observed. Results showed that CI patients were able to perform the linguistic task on the sung chords, but correct response times indicated sensory priming, with faster response times observed for the less-related targets: CI patients processed at least some of the pitch information of the musical sequences, which was stored in an auditory short-term memory and influenced chord processing. This finding suggests that the signal transmitted via electric hearing led to a pitch percept that was too different from that based on acoustic hearing, so that it did not automatically activate listeners’ previously acquired musical structure knowledge. However, the transmitted signal seems sufficiently informative to lead to sensory priming. These findings are encouraging for the development of pitch-related training programs for CI patients, despite the current technological limitations of the CI coding.

## Introduction

Satisfactory music perception, emotional, intentional prosody, and tonal language intelligibility remain barriers yet to be overcome by cochlear implant (CI) technology (e.g., Zeng, 2004; McKay, 2005; Gfeller et al., 2007). CIs are surgically implanted devices that directly stimulate the auditory nerve in individuals with profound deafness. However, while the current CI technology can restore speech perception in quiet for most users, the spectral information it is able to transmit is severely limited. One consequence of this limitation is that pitch perception remains very limited compared to normal-hearing (NH) listeners (see, e.g., Moore and Carlyon, 2005, for a review). Melody processing—a major component of music perception—requires some capacity for pitch processing. Various tests for music perception have been proposed to investigate CI users’ abilities to use the information provided by electric hearing. These tests include the assessment of listeners’ capacities in the discrimination of pitch changes and pitch direction, the identification of melodies and timbres, as well as the processing of rhythms and emotions (e.g., Cooper et al., 2008; Looi et al., 2008; Nimmons et al., 2008; Kang et al., 2009; Brockmeier et al., 2010; Gaudrain and Başkent, 2018; Zaltz et al., 2018). While rhythmic processing is close to normal, CI listeners have been shown to be impaired in tasks requiring pitch discrimination or pitch direction judgments, even though inter-subject variability can be large (for reviews, see McDermott, 2004; Moore and Carlyon, 2005; Drennan and Rubinstein, 2008). For example, pitch discrimination thresholds have been reported to vary from one or two semitones to two octaves, also as a function of frequency (Drennan and Rubinstein, 2008; Jung et al., 2010). Large variability has been also observed in impaired melodic contour processing, with performance ranging from chance level to close-to-normal performance (Galvin et al., 2007; Fuller et al., 2018). Melodic contour processing is also influenced by the timbre of the material (Galvin et al., 2008). When tested for familiar melody recognition and identification, CI listeners are impaired, but helped by rhythm or lyrics. CI listeners’ difficulties in recognizing familiar melodies are considerably enhanced when these are presented without lyrics or without the familiar rhythmic pattern.

Interestingly, the poor musical outcome may not only be due to CI technical limitations in transmitting pitch. Lack of training or negative attitudes to the new electric sound might also affect music perception (e.g., Trehub et al., 2009). Indeed, useable pitch information seems to be coded, given that training and exposure have been shown to provide improvements in music perception and appreciation (Leal et al., 2003; Lassaletta et al., 2008; Fuller et al., 2018). First, several reports have indicated correlations between self-reported listening habits, such as the amount of music listening, music enjoyment, and perceptual accuracy (e.g., Gfeller et al., 2008; Migriov et al., 2009). Second, several data sets suggest the possibility of training pitch perception in prelingually deaf children (Chen et al., 2010) and postlingually deaf adults (Fu and Galvin, 2007, 2008; Galvin et al., 2007). Training has also been shown to have beneficial effects on the recognition of musical instruments (Driscoll et al., 2009), on musical performance (Yucel et al., 2009; Chen et al., 2010) and on emotion recognition (Fuller et al., 2018).

In currently available studies on CI, pitch and music perception have been investigated with explicit testing methods requiring discrimination, identification, or recognition. These methods do not test for the implicit processing of pitch and music in CI listeners. However, implicit (indirect) investigation methods in various domains have been shown to be more powerful to reveal spared, preserved processing abilities than can be done by explicit investigation methods. For example, experiments using the priming paradigm have provided evidence for spared implicit processes despite impaired explicit functions in either visual or auditory modalities (Young et al., 1988 for spared face recognition in a patient with prosopagnosia; Tillmann et al., 2007 for spared music processing in a patient with amusia).

The priming paradigm investigates the influence of a prime context on the processing of a target event that is either related or unrelated. Its central feature is that participants are not required to make explicit judgments on the relation between prime and target, but to make fast judgments on a perceptual feature of the target (manipulated independently of the relations of interest). Such indirect, implicit tests may shed new light on our understanding of CI listeners’ music perception. Our present study tested music perception in CI listeners with a musical priming paradigm. This behavioral experimental method does not require explicit judgments and should be more sensitive to reveal spared pitch processing in these listeners, as suggested by effects of training and exposure.

In addition, while pitch discrimination thresholds in CIs are generally too large to detect the musically relevant difference of one semitone (e.g., Gaudrain and Başkent, 2018), combining multiple tones into a chord may yield different results. Indeed, the interaction of different pitch components, like in a chord, may result in spectro-temporal patterns in the implant that are more detectable than the variation of each of the components in isolation. In other words, while pitch representation in CIs is unlikely to resemble that in NH listeners, the representation of chords may be better preserved across modes of hearing (as suggested, for instance, by Brockmeier et al., 2011).

Measuring brain responses with the methodology of electroencephalography (EEG) can also provide some indirect, implicit evidence for music perception. Koelsch et al. (2004) reported musical structure processing in postlingually deafened, adult CI users who were not required to explicitly judge the tonal structure of musical sequences^1^. Event-related brain potentials (ERPs) were measured for musical events that were either expected (confirming musical structures and regularities, i.e., in-key chords) or unexpected (violating musical regularities, i.e., Neapolitan sixth chord^2^, also referred to as “irregular” chord). Furthermore, the unexpected, irregular chord was more irregular in the fifth position of five-chord sequences (thus in the final position) than in the third position. For control NH participants, the ERPs (in particular an early right anterior negativity, referred to as ERAN) were larger for the irregular chords than for the in-key chords, and even larger for the irregular chords in the fifth position than in the third position. It is thus not only the chord *per se*, but also its structural position in the sequence that raised the violation. For the CI participants, the irregular chords also evoked an ERAN, suggesting that the CI users processed the musical irregularity, even though the amplitudes of the ERAN were considerably smaller than in the NH control participants (leading to a missing ERAN in the third position). According to the authors, the observed ERP patterns indicated that the neural mechanisms for music-syntactic irregularity-detection were still active in CI patients. This finding suggests that CI listeners’ knowledge about the Western tonal musical system, which they had acquired prior to deafness, can be accessed despite the poor spectral signal transmitted by the CI. This finding was particularly encouraging for CI users as it indicated that their brains might accurately process music, even though explicitly CI users report difficulties in discriminating and perceiving musical information.

However, since the publication of this work, the domain of music cognition and neuroscience has advanced and pointed out that musical structure violations might introduce new acoustic information in comparison to the acoustic information of the context. The introduction of this new acoustic information provides an alternative explanation to musical irregularity effects based on sensory processing (instead of cognitive processing of musical structures) (see Bigand et al., 2006; Koelsch et al., 2007). Some of the musical violations used to investigate musical structure processing introduced new notes, which had not occurred yet in the sequence. These musical violations, which confounded acoustic violations and context effects, can be explained on a sensory level only. To confirm the influence of listeners’ musical structure knowledge (beyond acoustic influences), controlled experimental material is needed. This has been done in more recent behavioral and ERP studies in NH listeners (e.g., Bigand et al., 2001; Koelsch et al., 2007; Marmel et al., 2010), but for CI listeners, this experimental approach is still missing.

Our study fills in this need by testing postlingually deaf adult CI users with experimental musical material that allows the investigation of musical structure processing without acoustic confounds (i.e., the material used in Bigand et al., 2001). The musical sequences in our experiment were eight-chord sequences, with the last chord (i.e., the target chord) being either the expected, tonally regular tonic chord (i.e., related target) or the less-expected, subdominant chord (i.e., less-related target). A cognitive hypothesis predicted faster processing for the expected tonic than for the less-expected subdominant chord. To avoid acoustic confounds, neither the tonic nor the subdominant target occurred in the sequence. Furthermore, the experimental material was constructed in such a way to contrast this cognitive hypothesis of musical structure processing with a sensory hypothesis: Even though neither the tonic nor the subdominant target chord occurred in the sequence, the pitches of the component tones of the less-related subdominant target chord occurred more frequently in the sequence than those of the related tonic target chord. Consequently, faster response times for the less-related chord than for the related chord (thus the reversed pattern of the cognitive hypothesis) would point to sensory priming (also referred to as repetition priming): Sensory information would be simply stored in a sensory memory buffer, leading to facilitated processing of repeatedly presented information. This hypothesis does not require the activation of tonal knowledge, but is based solely on the acoustic features of the presented auditory signal. Alternatively, if the coding of the pitch information transmitted by the CI was too poor to lead either to cognitive or sensory priming, no difference in response times should be observed between the related and less-related targets.

To back up the cognitive and sensory explanations of our experimental material, we present three types of analyses of the experimental material. These analyses compared the related and less-related conditions for (1) the number of shared tones (pitch classes^3^) between target and prime context; (2) the overlap in harmonic spectrum; and (3) the similarity of acoustic information between the target and the prime context in terms of pitch periodicity (as in Koelsch et al., 2007; Koelsch, 2009; Marmel et al., 2010)^4^. While these analyses are originally designed to represent the normal auditory system, we also implemented versions of (2) and (3) through a simulation of electrical stimulation by a cochlear implant.

**FIGURE 1:**
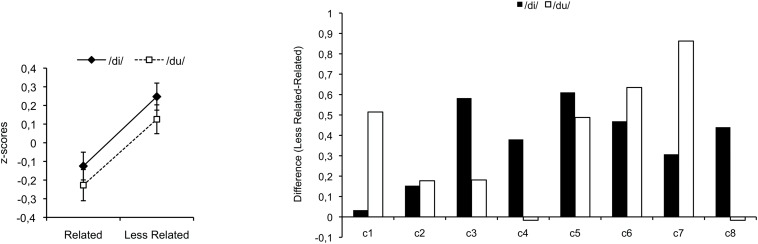
Average data of the 8 control participants in Tillmann et al. (2008) on the left, and their individual data on the right [this group of control participants had a mean age of 65 (±10) years]. For comparable data patterns of group of students, see Bigand et al. (2001) and Tillmann et al. (2008), of groups of children, see Schellenberg et al. (2005), and of another group of control participants as well as their individual data patterns, see Tillmann et al. (2007). Positive and negative values indicate facilitated processing for related and less-related targets, respectively.

In the present musical priming study investigating CI listeners, musical sequences, which ended on either the related tonic target or the less-related subdominant target (as described above), were presented as sung material, with a sequence of sung nonsense syllables (e.g., /ka//sha/etc). The last chord was sung on the syllable /di/ or /du/. Participants had to discriminate syllables by judging as fast as possible whether the last chord was sung on /di/ or /du/. This well-established musical priming implicit method allows measuring response times, supposed to reflect processing times of the last chord. For various populations of NH listeners, previous studies have shown the influence of tonal knowledge on processing speed and thus supported the cognitive hypothesis: Response times were faster for the related tonic chord than for the less-related subdominant chord. This result has been observed not only for English and French college students (musicians and non-musicians, Bigand et al., 2001; Tillmann et al., 2008), but also for 6-year-old children (Schellenberg et al., 2005), cerebellar patients (Tillmann et al., 2008), and amusic patients (Tillmann et al., 2007). Figure 1 represents this data pattern for the control group tested in Tillmann et al. (2008) (on the left) and for the individual participants (on the right), with positive values indicating faster response times for the related tonic chord than for the less-related subdominant chord. Note that we here plotted the individual data patterns from Tillmann et al. (2008) as this allows us to show the consistency of the priming pattern in control participants (particularly important as our present study did not include NH participants). Based on Koelsch et al. (2004) conclusions, we expected to observe the same data pattern for the CI users than previously observed for the NH users. The faster processing of the related tonic chord would indicate that the transmitted signal of the CI is sufficient to activate listeners’ musical knowledge acquired prior to deafness. The construction of our material is such that another pattern of results is also informative of the underlying processes. The reverse data pattern, where the less-related subdominant chord is processed faster, would indicate that the transmitted signal allows accumulation of sensory information in a short-term memory buffer, which then influences processing times (based on repetition priming). Finally, if the limited spectral resolution available through implants is not sufficient to provide relevant information to the CI user’s brain, then processing times should not differ between the two priming conditions.

## Materials and Methods

### Participants

Seven CI patients were tested in the present experiment using their own processor without their contralateral hearing aid. They were all postlingually deafened adult CI users who were implanted unilaterally (see Table 1 for participants’ characteristics, including information of the implant type, speech processor, coding strategy, and stimulation rate). Only one of the participants (ci7) reported having some musical training (9 years, starting at the age of 11, with 8 years of piano and 1 year of guitar), and reported currently practicing music about 1 h per week. All participants provided written informed consent to the study, which was conducted in accordance with the guidelines of the Declaration of Helsinki, and approved by the local Ethics Committee (CPP Sud-Est II). They were paid a small honorarium to thank them.

**TABLE 1:** Demographics of the seven participants.

**CI Participant**	**Gender**	**Age (years)**	**Hearing Loss**			**Implanted Ear**	**Duration CI use (years)**	**Contralateral hearing Aid**	**Implant**	**Speech processor**	**Strategy**	**Stimulation Rate**
			**Onset Time**	**Age at Deafness**	**Duration before implantation (years)**							
ci_1	F	68	Progressive	20	30	L	1	Y	CI24RE	Freedom SP	ACE 10 max	1200 Hz
ci_2	F	69	Progressive	25	15	R	1	Y	CI24RE	Freedom SP	ACE 12 max	1200 Hz
ci_3	F	47	Congenital and progressive	10	38	R	9	Y	CI24RE	Freedom SP	ACE 12 max	1200 Hz
ci_4	M	32	Early	56	20	R	2	Y	CI24RE	Freedom SP	ACE 10 max	1200 Hz
ci_5	F	32	Early	66	24	L	1	N	CI24RE	Freedom SP	ACE 10 max	900 Hz
ci_6	F	30	Progressive	25	11	L	1	Y	CI24M	Esprit 3G	CIS 12 channels	900 Hz
ci_7	F	33	Progressive	23	21	L	1	Y	CI24RE	Freedom SP	ACE 10 max	1200 Hz

*Onset time refers to the onset time of profound deafness (early: deafness begun in childhood, but after the development of spoken language (thus postlingually); congenital and progressive: the subject had a hearing loss at birth, but was hearing-aid fitted when 10-years old as the hearing-loss worsened).*

### Materials

The 48 chord sequences of Bigand et al. (2001) were used (with permission). These eight-chord sequences ended on either the tonally related tonic chord or the less-related subdominant chord (defining the target). The tonal relatedness of the final chords (the targets) was manipulated by changing the last two chords of the musical sequences (defining either a pair of dominant chord followed by tonic chord or a pair of a tonic chord followed by a subdominant chord). A further control was performed over the entire set of sequences, the material was constructed in such a way that a given chord pair (containing the target) defined the ending of the sequences in both related and less-related conditions. For example, when the first six chords (prime context) instill the key of C Major, the chord pair G-C functions as a dominant chord followed by a tonic chord. If, however, the prime context instills the key of G Major, the same chord pair functions as a tonic chord followed by a subdominant chord. Accordingly, over the experimental set, the 12 possible major chords served as related and less-related targets depending on the prime context.

Each of the first seven chords sounded for 625 ms and the target chord sounded for 1250 ms. The chord sequences were composed in such a way that the target chord never occurred in the sequence (see below for further analyses of the acoustic similarity between prime and target for the two experimental conditions). Example sequences are available as Supplemental Digital Content.

The sequences were sung on CV-syllables by sampled voice sounds (using Vocal-Writer software, Woodinville, WA, United States). Chords were generated by the simultaneous presentation of 3 or 4 synthetized utterances of the same syllable with different fundamental frequencies, which corresponded to the component tones of the chords. The succession of the synthetic syllables did not form a meaningful, linguistic phrase (e.g., /da fei ku ∫o fa to kei/), and the last syllable (i.e., of the target) was either /di/ or /du/ to define the experimental task. The experimental session consisted of 50% of sequences ending on the related tonic target (25% being sung with /di/, 25% with /du/) and 50% ending on the less-related subdominant target (25% with /di/, 25% with /du/). The experiment was run on Psyscope software (Cohen et al., 1993).

### Procedure

The sequences were presented over two loudspeakers (placed at about 80 cm in front of the participant, left and right from the screen of the laptop computer, thus at an azimuth of 45 degrees) at about 70 dB SPL, which was perceived as comfortable loudness level. The participants listened through the microphone of the processor using their everyday program and settings. The experiment was run in the main sound-field room of the University of Lyon CI clinic center.

The participants were asked to decide as quickly and as accurately as possible whether a chord was sung on /di/ or /du/ by pressing one of two keys. Incorrect responses were accompanied by an auditory feedback signal and a correct response stopped the sounding of the target. Participants were first trained on 16 isolated chords (50% sung on /di/ or /du/, respectively). The training phase was repeated in case the task was not understood, notably for difficulties to perceive the difference between the syllables or responding too slowly. Participants were encouraged to give their response while the target chord was still sounding, but a later time out was used (2800 ms) to not pressurize the participants too strongly. In the next phase of the experiment, the eight-chord sequences were explained to the participants with an example sequence and participants were asked to perform the same task on the last chord of each sequence. After four practice sequences, the 48 sequences were presented in random order twice in two blocks, separated by a short break. Two participants performed only one block (ci2, ci7). A short random-tone sequence was presented after each response to avoid carry-over effects between trials. The experiment lasted about 15 to 20 min.

## Auditory Properties and Perceptual Processing

When musical violations used to investigate musical structure processing introduce new tones, which had not occurred yet in the context, they confound the processing of acoustic violations with the cognitive processing of musical structures. The processing of these musical violations could thus be explained on a sensory level only, without the need for the involvement of listeners’ knowledge of musical structures (acquired prior to deafness). Experimental material must thus be constructed in such a way to control acoustic influences and disentangle them from cognitive influences (linked to listeners’ musical structure knowledge). In this section, we first present analyses of the acoustic and estimated perceptual similarities between the prime context and the related/less-related target in three ways, as previously done in the studies investigating NH listeners. We then present simulations that take in consideration potential changes due to the implication of the implant. None of the simulations predict facilitated processing for the related tonic chord in comparison to the less-related subdominant chord.

### Analyses of the Acoustic Similarity Between the Prime Context and the Target in Related and Less-Related Conditions

To check for acoustic influences, we analyzed our material in terms of (1) the number of pitch classes shared between targets and contexts as a function of the condition (related vs. less-related), (2) the spectral overlap of targets and contexts (simulating a place coding of pitch information), and (3) periodicity overlap in auditory short-term memory (simulating a temporal coding of pitch information). These analyses simulated different plausible pitch representations (place vs. time coding) and their integration over time. All analyses showed that acoustic influences would predict facilitated processing for the less-related subdominant chord. This prediction thus contrasts with cognitive, musical structure processing, which predicts facilitated processing for the related tonic chord.

#### Overlap in Pitch Classes Between Target and Context

To analyze the number of pitch classes shared between the target and the first seven chords (the context), we calculated (a) for each sequence, the number of occurrence of the target’s pitch classes in the first seven chords, and (b) the average over the sequence sets in the two conditions: The resulting mean was higher for the less-related condition (14.75 ± 2.05) than for the related condition (12.25 ± 2.93), *t*(11) = 2.61, *p* = 0.03. This finding thus represents a sensory advantage for the less-related subdominant targets and contrasts with the cognitive (tonal) advantage for the related tonic targets.

#### Spectral Contrast

**FIGURE 2:**
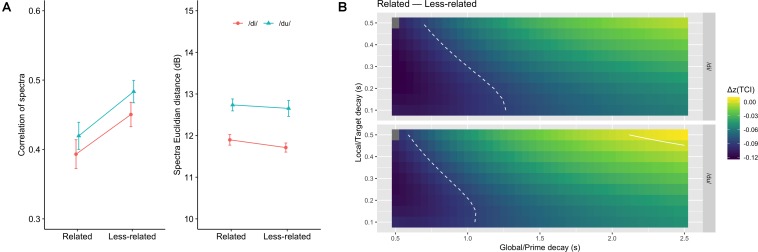
**(A)** Average spectral correlation (left) and Euclidian distance (right) for the related and less-related condition, and for the two target syllables. Higher correlations, and smaller Euclidian distances, are compatible with more sensory priming. **(B)** Difference in Fisher transformed tonal contextuality index between related and less-related conditions, as a function of syllable, and global and local decays. The solid line represents the value 0.0 where the two types of context have the same TCI. The dashed lines represent the critical limit beyond which differences can be considered significant.

To estimate the spectral overlap between target and context, we compared the spectra obtained from the first seven chords (of each sequence) with the spectra obtained from the corresponding target chord. The spectra were obtained by averaging the spectrogram computed over either the context or the target with FFT-time windows of 186 ms and 50%-window overlap (93 ms). Two metrics were used to judge the similarity of prime and target spectra: a correlation and an Euclidian distance. Because correlations are very sensitive to edge effects, the spectra were limited to the range 100 to 8000 Hz, and the overall spectral slope was compensated, in each sequence, based on the average of the target and prime spectra. The Euclidian distance was calculated on the spectrum expressed in decibels. Average correlation (Fisher-transformed, Fisher, 1921) and Euclidian distance values obtained for the sequence sets in the related and less-related conditions were then compared (Figure 2A). We analyzed those results with a repeated measures ANOVA with the factors syllable and relatedness. Correlation values were higher for the less-related condition than for the related condition [*F*(1,11) = 7.74, *p* < 0.05, ${\text{η}}_{\text{g}}^{2}$ = 0.20] thus confirming the acoustic advantage of the less-related subdominant chord. However, while the Euclidian distance did not significantly depend on the relatedness [*F*(1,11) = 0.75, *p* = 0.41,${\text{η}}_{\text{g}}^{2}$ = 0.02], it did depend on the nature of the target syllable [*F*(1,11) = 87.9, *p* < 0.001,${\text{η}}_{\text{g}}^{2}$ = 0.46]. All other effects and interactions were non-significant [*ps* > 0.22].

#### Pitch Periodicity in a Model of Auditory Short-Term Memory: Tonal Contextuality Index

To further test the acoustic influences in the experimental material, we used Leman (2000) model (as implemented in the IPEMtoolbox v1.02 by Leman et al., 2005) that stores auditory information in a short-term memory buffer. Acoustic input is first processed in a frontend module mimicking the peripheral auditory system (Van Immerseel and Martens, 1992). The output is then processed with a pitch module that extracts periodicities using an autocorrelation approach. Finally, the periodicity output is passed into a memory module. This model relies on the comparison of pitch images of two echoic memories, which differ in duration. With longer memory decays, the pitch images are smeared out, so that the images reflect the context echoic memory, while with a shorter decay the pitch images reflect the target echoic memory. Measuring the differences between the two images by computing their correlation gives an indication of how well the target (local) pitch image acoustically fits with the given (global) context. This measure is referred to as the Tonal Contextuality Index (TCI). In our case, to avoid choosing a specific point sample during the target to compare the periodicity patterns, we averaged them over the duration of the target, both for the short (local) and long (global) memory decays, and correlated these summary images. For the choice of the memory decay durations, as currently no precise information about the dynamics of auditory memory in human listeners are available, our simulations were carried out with local and global decay parameters varying systematically by steps of 0.05, from 0.1 to 0.5 s and from 0.5 to 2.5 s, for the local and global decay parameters, respectively, in order to explore a large parameter space of the model.

For the present analyses, the audio files of the 48 chord sequences were given as input to the model. TCI was calculated for each sequence and transformed into *z*-values using Fisher’s transformation (Fisher, 1921). The spaces of the differences between the TCI of the two targets are reported in Figure 2B for each target syllable. In these figures, positive Δ*z*(TCI) values indicate that the related condition yields stronger contextuality than the less-related condition, and negative values reflect the opposite. Here all Δ*z*(TCI) values were negative, indicating stronger TCI for subdominant targets than for tonic targets, thus predicting facilitated processing for the less-related targets. In Figure 2B, it can be seen that significantly negative values are found for short global decay values, but no significant positive values were observed. To further assess the role of relatedness on TCI, we analyzed the TCI data with a linear-mixed model with relatedness and syllable as fixed factors and tonality, local decay and global decay as random intercepts (*p*-values were obtained with Satterthwaite’s method). The model yields a significant effect of relatedness [*F*(1,17605) = 578.6, *p* < 0.0001], confirming that the less-related condition produced higher TCIs than the related one. The effect of syllable was also significant [*F*(1,17605) = 137.4, *p* < 0.0001] and so was the interaction [*F*(1,17605) = 5.78, *p* = 0.016]: the TCI was higher for /du/ than for /di/, but the that difference was less important in the less-related condition.

These results, thus confirm that the facilitated processing for tonic targets reported for NH participants by Bigand et al. (2001) and others (Schellenberg et al., 2005; Tillmann et al., 2007, 2008) reflect the influence of listeners’ knowledge about musical structures on target chord processing (see also Bigand et al., 2003).

In sum, the results of these three analyses confirmed that sensory and cognitive hypotheses make contrasting predictions for our experimental material: The sensory hypothesis predicts facilitated processing for the less-related targets, while the cognitive hypothesis predicts facilitated processing for the related targets. These predictions are in agreement with the alternative hypotheses made here above for the CI users: If the CI users show faster response times for the less-related subdominant chord, this finding would suggest the influence of the contextual auditory information (stored in a memory buffer) on target chord processing. If, however, CI users show faster response times for the related tonic chord, this finding would suggest the influence of CI users’ musical knowledge (acquired prior to deafness).

### Analyses of the Electrical Similarity Between the Prime Context and the Target in Related and Less-Related Conditions

In the previous sections, we examined the potential influence of sensory factors, rather than cognitive, on the priming effect induced by the material. Both the spectral contrast model (see section “Spectral Contrast”) and the tonal contextuality model (see section “Pitch Periodicity in a Model of Auditory Short-Term Memory: Tonal Contextuality Index”) assume a NH frontend. In the case of implant stimulation, the simultaneously presented tones that produce a chord interact and can generate other patterns. In other words, situations that would not induce sensory priming in NH listeners may very well do so in CI listeners.

To evaluate this possibility, we implemented a frontend mimicking a cochlear implant and the pattern of neural activation generated by electrical stimulation (Gaudrain et al., 2014). The model is based on the Nucleus Matlab Toolbox (Cochlear Ltd.), which generates patterns of electrical stimulation along the electrode array of the implant. The generated electrical field is then propagated in the cochlea to mimic current spread (Bingabr et al., 2008). Neural activation probability resulting from this electrical field is then calculated using approaches adapted from Rattay et al. (2001). This time-place image is used first to examine the spectral contrast between prime and target, and then to evaluate the tonal contextuality.

#### Spectral Contrast

**FIGURE 3:**
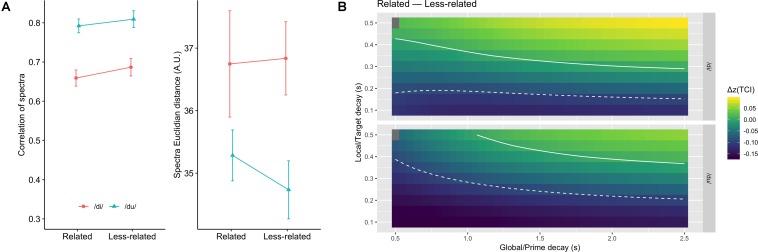
Same as Figure 2 for the CI model. **(A)** Average spectral correlation (left) and Euclidian distance (right) for the related and less-related conditions, and for the two target syllables. Higher correlations, and smaller Euclidian distances, are compatible with more sensory priming. **(B)** Difference in Fisher transformed tonal contextuality index between related and less-related conditions, as a function of syllable, and global and local decays. The solid line represents the value 0.0 where the two types of context have the same TCI. The dashed lines represent the critical limit beyond which differences can be considered significant.

As shown in Figure 3A, calculating the same metrics as in the section “Spectral Contrast,” i.e., correlation of detrended spectra, and Euclidian distance, again, there was no significant difference between the related and less-related conditions [for the correlation *F*(1,11) = 1.13, *p* = 0.31,${\text{η}}_{\text{g}}^{2}$ = 0.03; for the Euclidian distance *F*(1,11) = 0.18, *p* = 0.68,${\text{η}}_{\text{g}}^{2}$ = 0.003]. The nature of the syllable had a significant effect on both measures [for the correlation *F*(1,11) = 64.2, *p* < 0.001,${\text{η}}_{\text{g}}^{2}$ = 0.40; for the Euclidian distance *F*(1,11) = 19.0, *p* < 0.01,${\text{η}}_{\text{g}}^{2}$ = 0.17]. No interaction was significant.

#### Tonal Contextuality Index

The electrically induced neural activation image was used as auditory image to feed into the pitch module of the IPEMtoolbox in order to extract periodicity structures. The rest of the model (memory decay and TCI computation) was identical to the one used for the acoustic/NH model.

The results, shown in Figure 3B, indicate that no combination of local and global memory decay yields positive Δ*z*(TCI) beyond the critical value. When applying the same linear-mixed model as in the section “Pitch Periodicity in a Model of Auditory Short-Term Memory: Tonal Contextuality Index,” the effect of relatedness was found to be significant [*F*(1,35318) = 165.2, *p* < 0.0001], with the less-related condition having a globally higher TCI. The effect of syllable was also significant [*F*(1,35318) = 20.4, *p* < 0.0001] and so was the interaction [*F*(1,35318) = 48.6, *p* < 0.0001]: the TCI was higher for /du/ than for /di/, but the that difference was less important in the less-related condition.

From this analysis, it appears that, like for the NH model, the CI model predicts faster response times for the less-related condition than for the related condition.

## Results

**FIGURE 4:**
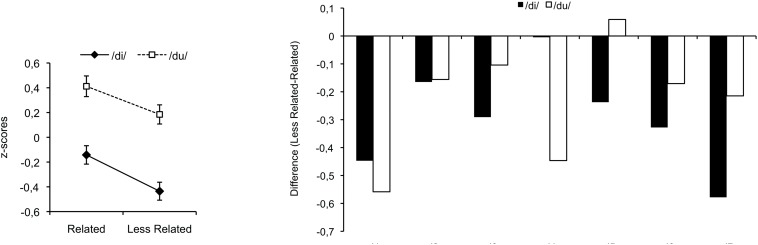
Average data of the CI participants on the left and their individual data on the right. Positive and negative values indicate facilitated processing for related and less-related targets, respectively.

Percentages of correct responses were high overall, with an average accuracy of 95% (ranging from 92 to 100%). Because of differences in average response latency between participants (ranging from 561 ms to 1567 ms) and with the goal to focus on differences between related and less-related targets, correct response times were individually normalized to *z*-scores with a mean of 0 and a standard deviation of 1 (Figure 4, left). *z*-Scores were analyzed with a 2 × 2 ANOVA with Musical Relatedness (related/less related) and Target Syllable (di/du) as within-participant factors. The main effect of Musical Relatedness was significant, *F*(1,6) = 23.32, *p* = 0.003, MSE = 0.02, indicating faster processing for less-related targets than for related targets. Overall, responses were faster for the syllable /di/ than /du/, *F*(1,6) = 19.26, *p* = 0.005, MSE = 0.13, as previously observed for NH listeners (Bigand et al., 2001). The interaction between Musical Relatedness and Target Syllable was not significant (*p* = 0.55).

Figure 4 (right) displays differences between less-related and related targets for the two target syllables for each participant. Positive values indicate faster processing for related targets, and negative values indicate faster processing for less-related targets. Faster processing for less-related targets was observed for all participants (except /du/ for ci5).

## Discussion

Our study investigated the perception of musical structures by postlingually deaf adult CI users. These listeners were mostly non-musicians without formal musical training. As shown in numerous studies in music cognition domain (e.g., Bigand and Poulin-Charronnat, 2006), NH non-musician listeners acquire implicit knowledge about the Western tonal musical system by mere exposure to music in everyday life and thanks to the cognitive ability of implicit learning (see also Tillmann et al., 2000). The postlingually deaf adult CI users participating in our present study had thus acquired this kind of implicit musical knowledge prior to deafness. We were investigating whether the signal quality of the CI—despite its poor spectral cues—is sufficient to activate this previously acquired implicit musical knowledge and to influence chord processing. We used a behavioral approach based on an indirect investigation method. The priming paradigm avoids asking for direct explicit judgments about the musical material, and takes advantage of implicit investigation. For music perception, the power of this implicit method has been previously shown by reporting musical structure knowledge in an amusic patient despite explicit music processing deficits (Tillmann et al., 2007) and in children as young as at the age of 6 years (Schellenberg et al., 2005), while explicit investigation methods had estimated the required age at 10 years (Imberty, 1981).

Based on our previous research, we used sung-chord sequences. The required priming task was a syllable-identification task (with syllables differing by one phoneme). The high accuracy we observed here showed that the CI users could perform this task without difficulty. The experimental material was constructed in such a way that three different data patterns could be expected, each indicating different underlying processes: (1) Faster processing of the related tonic chord would indicate that the transmitted signal of the CI is sufficient to activate listeners’ musical knowledge acquired prior to deafness; (2) faster processing of the less-related subdominant chord would indicate that the transmitted signal allows accumulation of sensory information in a short-term memory buffer, which then influences processing times (based on repetition priming); and (3) equal processing times for both chords would rather suggest that the limited spectral resolution available through implants is not sufficient to provide relevant information to the CI user’s brain.

The observed data pattern (see Figure 4) clearly supports the second hypothesis: Processing was facilitated for the less-related target, which is the target that benefited from sensory priming. This finding suggests that the signal transmitted via electric hearing was too different from the signal based on acoustic hearing, so that it did not automatically activate listeners’ previously acquired musical structure knowledge. However, the signal seems sufficiently informative to lead to sensory priming.

This conclusion seems to contradict the conclusion of Koelsch et al. (2004) based on ERPs in CI users, suggesting that tonal knowledge can be reached via electric hearing. We thus performed three acoustic analyses (pitch class, spectrum, pitch periodicity) also for the experimental material of Koelsch et al. (2004; see Supplemental Digital Content), notably to investigate acoustic similarities of the target with its preceding context. These analyses allowed us to resolve this contradiction. They showed that the material used by Koelsch et al. (2004) does not allow disentangling sensory and cognitive contributions in chord processing and the position effect. A more parsimonious explanation would thus be based on acoustic influences only. A similar argument has been made later by Koelsch et al. (2007), thus leading the authors to use new musical material with more subtle musical structure manipulations. However, this new material had not yet been used in an EEG study investigating CI users.

Our present findings support the hypothesis that despite the poor coding of spectral information, the CI transmits some pitch-related information of the musical material. Interestingly, based on our analyses, the observed sensory priming does not seem to be based on the individual pitches used, that is the spectral content *per se* (see section “Spectral contrast”). Instead, the sensory priming may be based on periodicity pattern similarity, as evidenced by the tonal contextuality index analysis (see section “Tonal Contextuality Index”). This is rather encouraging for implant users because, while their perception of pitch in natural sounds is very limited (e.g., in syllables, Gaudrain and Başkent, 2018 found discrimination thresholds for F0 between 4 and 20 semitones), it seems that when multiple tones are combined into chords, it makes periodicity patterns arise, to a degree that they can induce priming. This may suggest that, perhaps counter-intuitively, perception of chord sequences in CIs may be a more manageable goal than melody recognition. This finding thus integrates into other studies, suggesting that listeners can benefit from information provided by electric hearing even for music perception. Notably, studies have shown benefits of increased, self-imposed music exposure and of training programs to exploit the transmitted signal in music and pitch perception (e.g., Galvin et al., 2007). Converging evidence comes from the music appraisal of prelingually deaf CI listeners who are missing the comparison with music perception based on acoustic hearing (e.g., Trehub et al., 2009). Prelingually deaf children seem to find music interesting and enjoyable (Trehub et al., 2009; Looi and She, 2010) and children’s engagement with music may also enhance their progress in other auditory domains (Mitani et al., 2007; Rochette and Bigand, 2009). Galvin et al. (2009) have shown a high variability in melodic contour identification across CI users, with some musically experienced CI users performing as well as NH listeners. Most importantly, they showed that training on melodic contour (using visual support) improves performance in melodic contour identification in CI users, even though the transmitted signal was not changed. It is worth further clarifying that with “pitch” we are here referring to the acoustic information that is related to the pitch percept in NH listeners. Indeed, we need to acknowledge that we do not know whether this is a subjective pitch percept for the CI listeners, as it is for NH listeners, but can ascertain it is related to that acoustic information.

Our findings together with other data sets on the beneficial effects of musical training and musical exposure are thus encouraging for the development of pitch-related training programs for CI patients. Indeed, in parallel to the technological efforts aiming to improve the coding strategies implemented in the CI device, efforts need to be made for training programs in order to improve how the brains of CI users are exploiting the transmitted signal.

Training programs might need to work differently for postlingually deaf adult CI users and prelingually deaf child CI users. Because of the differences between acoustic and electric hearing, adults find music often disappointing or unacceptable, leading to changes in listening habits and decreased subjective enjoyment in comparison to prior to deafness (McDermott, 2004; Lassaletta et al., 2008; Looi and She, 2010). In contrast, the child implant users find music interesting and enjoyable (Trehub et al., 2009; Looi and She, 2010). The postlingually deaf adult CI users have acquired cognitive patterns and schemata for speech and music based on their previously normal (or impaired, but aided) hearing. However, the information provided by the CI is different, in particular for the coding of the spectro-temporal fine structure. The prior knowledge, which was developed based on the information available in acoustic hearing, might thus create perceptual filters and cognitive schema of “pitch” in music, which result in costs for picking out the relevant information from the transmitted signal. In contrast to the postlingually deaf adult CI users, early-implanted children CI users have developed their speech and music patterns based on the information in the electric hearing. As they are missing the comparison to the information provided by acoustic hearing, they appreciate and enjoy music, while for the postlingually implanted adults the CI version of music is only a poor representation of their memory. Training programs would thus need to increase exposure leading to the construction of newly shaped perceptual filters and schemata. The findings of our present study provide some further grounds for this training by showing that some pitch information is implicitly processed by the adult CI users. They thus have implications for rehabilitation programs, notably by encouraging training strategies that rely on spared implicit processing resources.

## Data Availability

The datasets generated for this study are available on request to the corresponding author.

## Ethics Statement

All participants provided written informed consent to this study, which was conducted in accordance with the guidelines of the Declaration of Helsinki, and approved by the local Ethics Committee (CPP Sud-Est II).

## Author Contributions

BT, BP-C, IA, and LC contributed to the conception and design of the study. BT, BP-C, and IA acquired the data. BT, BP-C, EG, and CD performed the statistical analysis and modeling. ET and LC provided access to the patients and related information. BT wrote the first draft of the manuscript. EG and CD wrote the simulation sections of the manuscript. All authors contributed to the manuscript revision, and read and approved the submitted version.

## Conflict of Interest Statement

The authors declare that the research was conducted in the absence of any commercial or financial relationships that could be construed as a potential conflict of interest.

## Figures and Tables

**APPENDIX FIGURE 1:**
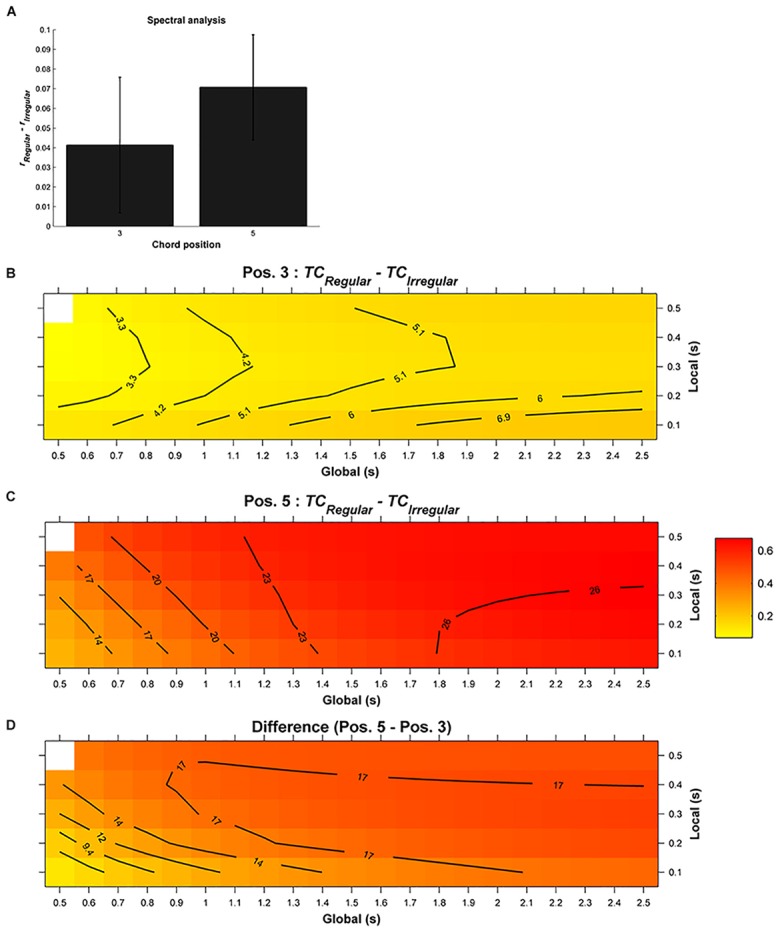
Results of the acoustic analyses for the material Koelsch et al. (2004), presenting differences between the regular and irregular chords for positions 3 and 5, for the harmonic spectrum **(A)** and the model of Leman **(B–D)**. For panels B and C, the mean differences between the tonal contextuality of the target chords (TC_regular_ – TC_irregular_) are presented as a function of the local and global context integration windows. Positive, negative, and non-significant differences are represented by hot (i.e., red), cold (i.e., blue), and white colors, respectively (two-tailed paired *t*-test, *p* < 0.05; *t*-values are reported as contours). A positive difference indicates that the pitch similarity in the sensory memory induced by the related target is stronger than that of the less-related target. Panel D completes this presentation with the difference for positions 5 and 3.

## Footnotes

1 The sequences also contained timbral deviants and participants were required to detect these deviants (65% of correct responses for the CI users vs. 79% for the control group). CI listeners’ event-related brain potentials also reflected the detection of the timbre change, even though with smaller amplitude than NH control participants. As the timbres of the used instruments (piano, organ) also differed by their temporal attack, behavioral and neural responses to the timbre change (indicating acoustic irregularity detection) might be based on these temporal envelope changes, in addition to the spectral changes.

2 The “Neapolitan sixth chord” is a variation of the subdominant chord (an in-key chord), but contains two out-of-key notes (in the tonality of *C* major, these are the notes *a*♭ and *d*♭ in the chord: *a*♭ – *f* – *d*♭). When this chord is used as a substitution of a subdomimant chord, it sounds unusual, but not wrong (as in the third position of the sequences by Koelsch et al., 2004). However, when used as a substitution of a tonic chord, it sounds wrong (as in the fifth position of the sequences by Koelsch et al., 2004).

3 A pitch class is defined as a set of pitches at different pitch heights that are separated by octaves (i.e., an interval defined by multiples of the fundamental frequency of a tone), for example the tones at 220, 440, and 880 Hz all belong to the pitch class of A.

4 For the experimental material of Koelsch et al. (2004), these three analyses are presented as Supplemental Digital Content.

## Appendix

### Analyses of the Acoustic Similarity Between the Harmonic Context and the Target in Regular and Irregular Conditions of Koelsch et al. (2004)

We ran the three analyses investigating the acoustic similarity between contexts and targets also with the material of Koelsch et al. (2004), which is currently the only paper that tested musical structure processing in CI patients with an indirect investigation method. A total of 260 tonal harmonic sequences made up of five chords were generated as a MIDI file from the authors’ description.^5^ An audio file was then synthetized using a piano timbre. Duration of the first four chords was 600 ms, while the fifth chord sounded for 1200 ms. Sequences were presented in six blocks to the model. Each block was in a different tonal key (*C*, *D*, *E*, *F#*, *Ab*, and *Eb* keys). To conform to the experimental procedure reported by the authors, echoic memory state was reset between each block, but not between each sequence within a block.

### Overlap in Pitch Class Between Targets and Contexts

We analyzed the number of pitch classes shared between a target chord and its preceding chords, as a function of target type and target position within the sequence. We used the MIDI file generated as described above. The mean number of pitch classes shared between target and preceding context was higher for the tonic regular condition (5.4 ± 3.29) than for the irregular condition (1.35 ± 1.34). More interestingly, the difference between regular and irregular targets was stronger for the fifth position (6.41) than for the third position (1.68), revealing a sensory advantage for the regular targets at the fifth position. Consequently, the cognitive (tonal) advantage for regular targets over irregular targets was confounded with acoustic similarity.

### Spectrum Analyses

We estimated the spectral overlap between the target chords and their respective harmonic contexts. For each sequence of the material of Koelsch et al. (2004)’s study, the spectrum obtained from the target chord was correlated with the spectrum obtained from preceding chords. The spectra were obtained by averaging the spectrogram computed over the contexts and targets with FFT-time windows of 186 ms and 50%-window overlap (i.e., 93 ms). A Position (3 vs. 5) × Chord Type (Regular vs. irregular) ANOVA on average correlation values showed not only main effects of both Position, *F*(1,256) = 308.51, MSE = 0.175, *p* < 0.0001, and Chord type, *F*(1,256) = 359.82, MSE = 0.204, *p* < 0.0001, but—most importantly—a significant interaction, *F*(1,256) = 24.65, MSE = 0.014, *p* < 0.0001: The difference between regular and irregular chords was stronger for position 5 than for position 3, *t*(64) = 5.55, *p* < 0.0001 (see Appendix Figure 1A here below), thus confirming a sensory advantage at position 5.

### Pitch Periodicity in Auditory Short-Term Memory

We here ran the implementation of Leman (2000) model and its IPEM toolbox as used in Bigand et al. (2014) and its graphical presentation of the local and global parameters and Tonal Contextuality index (TC). For the material of Koelsch et al. (2004), the differences between regular chords (i.e., in-key) and irregular chords (i.e., Neapolitan sixth chord) are reported in Appendix Figures 1B,C here below, for positions 3 and 5, respectively. Irrespective of position within the sequence, irregular chords elicited a stronger dissimilarity in short-term pitch memory than did regular, in-key target chords (as illustrated by the hot colors, TC of the in-key chords were higher than for the irregular chords, i.e., TC_in–key_ – TC_irregular_ > 0). In addition, the size of the dissimilarity mirrored the size of the ERAN, thus accounting for the effect of position: Simulations predicted stronger dissimilarity for the irregular chords occurring at the syntactically incorrect position (i.e., at position 5; Appendix Figure 1C), compared to the syntactically correct position (i.e., at position 3; Appendix Figure 1B). The stronger dissimilarity of the target at position 5 in terms of TC is illustrated in Appendix Figure 1D that reports a (positive) difference between position 5 minus position 3. In sum, the sensory model accounts for the data pattern observed in Koelsch et al. (2004), suggesting that in this study the effect of position in participants’ data might have a sensory rather than a cognitive origin.

To summarize these analyses, for the experimental material used in Koelsch et al. (2004), cognitive and sensory hypothesis made the same predictions, that is, a cost of processing is expected for the musically irregular chord, in particular in the fifth position. The three types of analyses thus reveal that we cannot conclude whether CI participants’ ERPs reflected the violation of information accumulated in a sensory memory buffer or the activation of musical knowledge (see Koelsch et al., 2007, for a similar argument for this experimental material in NH listeners).
